# Determining the Persistence of Xylazine and Ketamine in Cattle Tissue Following a Simulated Rendering Process

**DOI:** 10.3390/vetsci12080740

**Published:** 2025-08-07

**Authors:** Scott A. Fritz, Michael D. Kleinhenz, Steve M. Ensley, Patrick J. Gorden, Yuntao Zhang, Johann F. Coetzee, Michael D. Apley

**Affiliations:** 1 Department of Anatomy and Physiology, Kansas State University College of Veterinary Medicine, Manhattan, KS 66506, USA; scottfritz@vet.k-state.edu (S.A.F.);; 2 Veterinary Education, Research and Outreach (VERO), Texas A&M University, Canyon, TX 79015, USA; 3 Department of Veterinary Diagnostic and Production Animal Medicine, Iowa State University, Ames, IA 50011, USA

**Keywords:** drug residues, euthanasia, ketamine, xylazine, rendering

## Abstract

Humane euthanasia is an endpoint for production animals succumbing to disease or trauma. Euthanasia performed with barbiturates or other anesthetic/sedative drugs observes zero withdrawal time, and drug residues may remain in tissues. Carcasses may be submitted for rendering, and rendered products can be used to manufacture pet foods. The purpose of this study was to determine the concentration of two drugs, xylazine and ketamine, that may be used during the euthanasia process of food animals and to determine the fate of these drugs following a simulated rendering process using a commercial autoclave. Twelve cattle were administered xylazine or xylazine and ketamine prior to euthanasia via penetrating captive bolt, and samples of muscle, fat, liver, and kidney were collected. The tissue samples were analyzed by LC-MS/MS, both raw and following rendering. The parent compounds xylazine and ketamine were detected in all tissues, both before and after rendering. The highest concentrations were found in rendered kidney for both drugs, and the lowest in rendered and raw fat for xylazine and ketamine, respectively.

## Simple Summary

Humane euthanasia is an endpoint for production animals succumbing to disease or trauma. Multiple methods of humane euthanasia are approved by the American Veterinary Medical Association (AVMA), including the administration of barbiturates alone or administering anesthetic drugs prior to physical methods, including penetrating captive bolt or gunshot. Carcasses of deceased animals can be submitted for rendering, and rendered products can be used to manufacture pet foods. Barbiturate drugs are known to persist in tissue and survive rendering, so alternative options for euthanasia are desirable. Xylazine and ketamine are two drugs that may be used during euthanasia. The distribution of these drugs immediately to tissue and their fate following rendering is unknown. Cattle were administered these drugs as part of the euthanasia process, and their concentrations were determined in raw tissue and tissue exposed to a simulated rendering process. The parent compounds xylazine and ketamine were detected in all tissues both before and after the rendering process. Future confirmatory research using a large-scale commercial rendering facility is warranted.

## 1. Introduction

Humane euthanasia is an endpoint for some food animals experiencing disease or trauma. Multiple methods of euthanasia are approved by the American Veterinary Medical Association (AVMA) for use in bovids and small ruminants, including the following: barbiturates and barbituric acid derivatives alone or physical methods, like gunshot and penetrating or non-penetrating (calves) captive bolt, together with adjunctive methods, like exsanguination, pithing, or IV injection of potassium chloride [1]. Carcass management is an important consideration of euthanasia. Some carcasses can be submitted for a process known as rendering following euthanasia. According to the United States Department of Agriculture (USDA), “rendering is an off-site process that uses heat to convert animal carcasses into safe, pathogen-free feed protein and other valuable end products while reducing the negative effects of the carcasses on people and the environment [2].” The rendering industry in the United States is dominated by tube and disc continuous rendering systems [3]. In these systems, raw material at ambient temperature is fed, on a conveyor, into the disc or tube cooker where it is cooked at 240–280 °F (~115–145 °C) for 30–40 min [3]. Moisture evaporates, and liquid fat is separated from the solids following cooking. The solids go through a pressing process that removes more fat, and the fat and solid portions continue on separate pathways.

Animals are often chemically restrained with sedative or anesthetic drugs for safety when using physical methods of euthanasia. Unfortunately, there are instances where sodium pentobarbital-treated animal tissues have been consumed as raw or contaminated animal food products, resulting in severe clinical outcomes in animals that consume these products [4,5,6,7]. Sodium pentobarbital has been shown to resist degradation during rendering [8]. Alternative drug options to chemically restrain animals during euthanasia that do not pose a risk to animals consuming either the raw or rendered tissues of the euthanized animal are desirable. Xylazine and ketamine are drugs that may be used for chemical restraint during the euthanasia process.

Xylazine is an alpha-2 agonist with analgesic, muscle relaxation, and sedative effects. Ketamine is a dissociative anesthetic commonly used for chemical restraint, anesthesia, and pain management. Xylazine and ketamine are described as being highly lipophilic and consequently have large volumes of distribution, indicating they widely distribute throughout the body [9,10,11]. This rapid distribution into the tissues includes the central nervous system, contributing to the rapid clinical effects. These compounds are used to make animals calmer and easier to restrain in order to perform euthanasia, often via penetrating captive bolt or gunshot, where animals need to remain still.

While some pharmacokinetic data exist for animals administered these drugs, there are no data on the tissue concentrations of xylazine or ketamine in cattle tissue immediately after administration [9,10]. Anesthetic drugs are typically considered low-risk compounds for residues in food products due to their typical short-term use, short half-lives, often intravenous administration, low doses needed for the desired effect, and more veterinary oversight compared to some antibiotic compounds [12]. The Food Animal Residue Avoidance Database (FARAD) suggests meat withdrawal intervals of 3 and 5 days for ketamine and xylazine, respectively [13,14]. These withdrawal times apply to animals harvested for human consumption. Both ketamine and xylazine have been detected in tissues collected from horses euthanized after utilizing these drugs for chemical restraint [15]. The concentration of xylazine and ketamine in cattle tissue immediately following administration, as in the case of sedation for euthanasia, is unknown. Cattle are more sensitive to xylazine than horses, and smaller doses are used to achieve the same effect [12]. Currently, the United States Department of Agriculture Food Safety and Inspection Service (USDA-FSIS) tests for ketamine and xylazine as part of its multi-drug screening and confirmation assay [16]. The listed applicability levels for both ketamine and xylazine in kidneys and muscles are 20 ng/g and 1 ng/g, respectively [16], for the screening assay. The confirmation assay lists applicability levels for ketamine at 20 ng/g in muscles and kidneys, while xylazine is listed as “N/A”.

While xylazine and ketamine are typically administered intravenously for the purpose of restraint, the exposure in animals consuming the rendered products from treated animals is primarily oral. Oral bioavailability data for these drugs have not been fully described in all species. Cats administered 2.5 mg/kg xylazine and 10 mg/kg ketamine per os concurrently exhibited sedative effects similar to intramuscular administration of the same combination, suggesting active drugs are absorbed systemically following ingestion [17]. In humans, extensive first-pass metabolism of ketamine occurs in the liver following oral administration, resulting in a low oral bioavailability calculated at 17% [18].

The stability of some veterinary drugs in cooked beef or after exposure to various levels of environmental heat has been reported [19,20,21,22]. Changes in the concentrations of some veterinary drugs in milk products have been shown to significantly increase or decrease depending on the process used to produce the products [23]. However, the persistence of xylazine and ketamine following rendering is unknown, which creates a significant data gap in recommending their use in euthanasia protocols for food animals. If these drugs are destroyed by the rendering process, they could be used to chemically restrain food animals during the euthanasia procedure with little risk of inadvertent exposures through rendered products.

### Objectives

To address potential food safety issues of the use of xylazine and ketamine for sedation in association with food animal euthanasia, this study had two objectives. (1) Determine the tissue concentrations of xylazine and ketamine in tissues of cattle immediately following administration of these drugs as part of the euthanasia procedure. (2) Determine the persistence of these drugs in cattle tissue following a simulated rendering process.

## 2. Materials and Methods

Tissues for this project were procured from animals from previously completed research projects (KSU IACUC #-4937, ISU IACUC #-22-217). Group one consisted of six 10–14-week-old beef/dairy crossbred calves that weighed 59–68 kg and were intravenously administered 0.2 mg/kg xylazine prior to penetrating captive bolt euthanasia. Group two consisted of six adult female lactating Holstein cows that weighed 660–868 kg and received intravenous ketamine (2 mg/kg) and xylazine (0.5 mg/kg) mixed together in a syringe prior to penetrating captive bolt once the cows reached an unconscious state. For both treatment groups, the samples collected included liver, kidney, muscle, and fat from each individual animal. The muscle samples were collected from the semimembranosus and semitendinosus muscles of the hind limbs. Perirenal fat was collected from group two, while the fat samples from group one were collected from multiple locations, including the perirenal fat, to obtain enough sample weight. The animals were euthanized, and the tissue samples were collected and frozen at −80 °C until analysis. The samples were thawed on a benchtop at room temperature, and 10 subsamples for each animal were created, including the following:

1. RAW—individual tissues (fat, liver, muscle, kidney) not subject to heating prior to drug analysis.
2. RENDER—individual tissues (fat, liver, muscle, kidney) subject to heating prior to drug analysis.
3. RAW–COMP—all tissues combined prior to drug analysis.
4. RENDER–COMP—all tissues combined prior to heating and drug analysis.

For the rendered samples, 20 g of each tissue was weighed into glass beakers and sealed with aluminum foil. For the combined sample, 5 g of each of the four tissues was weighed into a beaker for a total of 20 g of tissue. All the rendered samples were rendered as one piece of tissue to mimic the rendering process, with the exception of the combined samples, which were composed of four pieces weighing five grams each. All the rendered samples were processed on a single autoclave run. For the raw samples, 2 g of each tissue was weighed into 50 mL conical tubes. The combined raw sample included 2 g of each sample type. The raw samples were homogenized and analyzed without further treatment.

### 2.1. Rendering

To mimic the continuous rendering process most often used in the US, a commercial autoclave (Model: Lancer LSS 275, Getinge, Wayne, NJ, USA) was utilized. A custom cycle was created to achieve temperatures of 240 °F (115.56 °C) for 30 min at atmospheric pressure. The autoclave run lasted for 33:09 min at 240 °F, with a minimum temperature of 240.5 °F (115.83 °C) and a maximum temperature of 242.1 °F (116.72 °C). The temperature was monitored by the autoclave system every minute of the run. Following the autoclave procedure, the samples were homogenized and submitted for analysis via UPLC-MS/MS.

### 2.2. Drug Analysis

Drug analysis was adapted from previously described work analyzing the same compounds in similar matrices [24,25].

#### 2.2.1. Chemicals and Reagents

Pharmaceutical standards ketamine, ketamine-d4, xylazine, and xylazine-d6 were purchased from Sigma-Aldrich (St. Luis, MD, USA). LC/MS-grade methanol and acetonitrile were purchased from Fisher Scientific (Waltham, MA, USA). LC/MS-grade formic acid was purchased from Fisher Scientific (Waltham, MA, USA).

#### 2.2.2. Stock Solutions

The ketamine and xylazine stock solutions were prepared in methanol at 1 mg/mL and stored in a −80 °C freezer. The ketamine-d4 and xylazine-d6 were prepared in methanol at 100 µg/mL and stored in a −80 °C freezer.

#### 2.2.3. Standard Working Solutions

The standard stock solutions were moved out of the −80 °C freezer and thawed to room temperature on a benchtop before use (20 min). For daily analysis, the ketamine and xylazine stock solutions were diluted with methanol to obtain a mixture of ketamine and xylazine at 10 µg/mL. Subsequently, the mixture of ketamine and xylazine was further diluted to obtain a set of concentrations at 2.5, 5, 10, 25, 50, 100, 250, 500, 1000, 2500, and 5000 ng/mL in methanol.

#### 2.2.4. Internal Standard Working Solutions

The internal standard stock solutions were moved out of the −80 °C freezer and thawed to room temperature on a benchtop before use (20 min). For daily analysis, the ketamine-d4 and xylazine-d6 stock solutions were diluted with methanol to obtain a mixture of ketamine-d4 and xylazine-d6 at 1 µg/mL.

#### 2.2.5. Sample Preparation

A total of 2 g of each homogenized tissue sample was put into a 50 mL centrifuge tube, and 2 g of anhydrous sodium sulfate was added. A volume of 100 µL of an internal standard working solution (1 µg/mL) was added. The samples were extracted with 10 mL of ammonia-modified acetonitrile (5%, *v*/*v*). After vortexing with a multiple-tube Vortexer (FischerBrand™, Fischer Scientific, Waltham, MA, USA) at 2000 rpm for 30 min, the samples were centrifuged at 3500 rpm for 10 min at 4 °C. A volume of 3 mL of the supernatant was transferred into a 15 mL centrifuge tube. A volume of 1 mL of hexane was added to the supernatant to remove the remaining fat matrix. After vortexing with a multiple-tube Vortexer at 2000 rpm for 30 min, the samples were centrifuged at 3500 rpm for 10 min at 4 °C. A volume of 2 mL of the lower layer fraction was transferred into a glass tube and concentrated by drying at 45 °C until dry. The dried sample was reconstituted in 0.2 mL of 0.1% formic acid/acetonitrile (30/70) and vortexed for 30 s. The reconstituted solution was passed through a 0.22 µm filter by centrifuging at 13,000 RPM for 10 min at 4 °C and analyzed by UPLC-MS/MS.

#### 2.2.6. Instrumentation

The analysis was carried out by UPLC–MS/MS utilizing a Waters Acquity Ultra-Performance LC with a Waters column manager and heater/cooler, binary system manager, and sample manager coupled to a Waters Xevo TQ-S triple–quadrupole mass spectrometer equipped with electrospray ionization (Waters Acquity Ultra Performance LC, Xevo TQ-S MS/MS, Waters Co., Milford, MA, USA). A Waters Acquity UPLC BEH C18 Column (130 Å, 1.7 µm, 2.1 mm × 100 mm) was utilized, combined with eluents composed of mobile phase A (0.1% formic acid in Milli-Q water) and mobile phase B (acetonitrile). The flow rate was 0.4 mL/min. The gradient program was as follows: from 0 to 1 min, phase A/phase B at 82%/18%, respectively; at 3 min, 65%/35%; and at 3.01 min, 82%/18% for 1.99 min. The total run time was 5 min. The MS/MS ionization mode used was positive electrospray ionization. The operating parameters for the mass spectrometer were as follows: the capillary voltage was 3.70 kV, and the source and desolvation temperatures were 150 °C and 600 °C, respectively. The cone energy was set at 16 V. Nitrogen was used as the desolvation gas and cone gas. Helium was used as the collision gas. The collision gas flow was 0.15 mL/min. The desolvation and cone gas flows were 900 and 150 L/h, respectively. Data analysis and quantification were performed using Waters MassLynx and TargetLynx software 4.1, respectively.

#### 2.2.7. Method Validation

The developed method was validated in accordance with selectivity, limit of detection (LOD), limit of quantification (LOQ), linearity, accuracy, and precision.

Selection and tuning of the parent and daughter ions, as well as analyte-dependent parameters, such as collision energy and Dwell, were performed by combined infusion of individual pharmaceutical solutions at a concentration of 1000 ng/mL in 0.1% of formic acid/acetonitrile (30/70). The mass spectra for the ketamine, ketamine-d4, xylazine, and xylazine-d6 were obtained individually. The selection of the parent ion for each analyte was accomplished. These parameters were later used to constitute the Multiple Reaction Monitoring (MRM) method as follows. The quantification was conducted using the MRM mode with transitions at *m/z* 238.0938 → 179.1188 (qualifier) and *m/z* 238.0938 → 125.1085 (quantifier) for ketamine and *m/z* 242.1776 → 129.0691 (quantifier) and *m/z* 242.1776 → 183.1397 (qualifier) for ketamine–d4 (internal standard). For xylazine, the quantification was conducted using the MRM mode with transitions at *m/z* 221.149 → 164.0811 (qualifier) and *m/z* 221.149 → 90.0736 (quantifier) for xylazine and *m/z* 227.149 → 90.0765 (quantifier) and *m/z* 227.149 → 170.1366 (qualifier) for xylazine-d6 (internal standard).

To prepare calibration standard curves, a volume of 100 µL of each standard working solution and 50 µL of an internal standard working solution were added into 850 µL of a 0.1% formic acid/acetonitrile solution (30/70) to obtain a set of standard concentrations at 0.25, 0.5, 1, 2.5, 5, 10, 25, 50, 100, 250, and 500 ng/mL. The internal standard concentration was 50 ng/mL. To prepare QC samples, 2 g of blank bovine muscle, fat, liver, and kidney were analyzed with no alteration. Positive control samples were spiked with 200 ng ketamine and xylazine standards to obtain concentrations of ketamine and xylazine at 100 ng/g; then, 100 ng of ketamine-d4 and xylazine-d6 was spiked to obtain concentrations of ketamine-d4 and xylazine-d6 at 50 ng/g so that the internal standards in the calibration standard solutions, the QC samples, and the test samples were at the same concentration of 50 ng/g. Negative QC samples were analyzed adjacent to their spiked counterparts. For ketamine, the calibration curve was linear from 0.25 to 500 ng/mL and was accepted with a correlation coefficient R^2^ of at least 0.99. For xylazine, the calibration curve was linear from 0.25 to 500 ng/mL and was accepted with a correlation coefficient R^2^ of at least 0.99. For both compounds, the LOD and LOQ were 0.1 and 0.25 ng/mL, respectively.

The inter-day accuracy and precision were assessed by analyzing 2 replicated QC samples at the level of 100 ng/g on three separate days. The recovery ratio (%) and relative standard deviation (RSD%) were used to evaluate accuracy and precision. For xylazine spiked in bovine muscle, fat, liver, and kidney, the accuracy was 99.98%, 99.62%, 100.52%, and 100.87% at a concentration of 100 ng/g, respectively. The inter-day precision was 4.29%, 1.28%, 0.17%, and 1.75% at a concentration of 100 ng/g, respectively. For ketamine spiked in bovine muscle, fat, liver, and kidney, the accuracy was 103.88%, 109.60%, 106.72%, and 105.63% at a concentration of 100 ng/g, respectively. The inter-day precision was 1.39%, 3.44%, 1.50%, and 1.04% at a concentration of 100 ng/g, respectively.

## 3. Results

### 3.1. Tissue Concentrations

Table 1 summarizes the individual tissue concentrations of xylazine and ketamine. Graphical representation of the same data is available in the Supplementary File. The kidney tissue contained the highest concentration of xylazine in all individuals, regardless of the treatment group. The liver and muscle tissues contained similar xylazine concentrations, followed by the fat tissue, with relatively low concentrations. Group 1 received 0.2 mg/kg xylazine IV, and the highest detected kidney concentration was 1562 ng/g in the raw tissue and 2033.5 ng/g in the rendered tissue. Group 2 received 0.5 mg/kg xylazine IV, and the highest detected kidney concentration was 4093.6 ng/g in raw tissue and 6866.8 ng/g in rendered tissue. Ketamine was found in the highest concentrations in the kidney tissue, followed by the muscle, liver, and fat tissues. Group 2 received 2 mg/kg ketamine IV, and the highest kidney concentration was 3321.3 ng/g in the raw tissue and 4528.2 ng/g in the rendered tissue. The rendered tissue contained higher drug concentrations than the raw tissue counterpart, with the exception of xylazine in fat.

### 3.2. Tissue Concentration Changes

The percent change in drug concentrations for each treatment group is presented in Table 2. Nearly all analyzed samples exceed the USDA-FSIS applicability concentrations of xylazine (20 ng/g) and ketamine (1 ng/g) in animals euthanized immediately following administration.

### 3.3. Moisture Loss

The sample weight reductions following rendering are presented in Table 3. All sample types were reduced by similar degrees, with the exception of the composite samples.

## 4. Discussion

Tissue concentrations of xylazine were highest in the kidney tissue from both treatment groups, consistent with the reported primary urinary excretion and short half-life [9]. Group 2 had higher overall xylazine concentrations compared to group 1, consistent with the larger dose administered. Ketamine was also highest in the kidney tissue, but the liver and muscle tissues contained similar concentrations, while the fat tissue contained less. The mean raw muscle xylazine concentrations were 140.35 ng/g and 304.07 ng/g for treatment groups one and two, respectively. The concentrations were similar to previous studies that detected 103.1–112.4 ng/g xylazine in the muscle of horses following euthanasia [15]. The mean muscle ketamine concentration in the present study was 1078.02 ng/g, which is similar to the 1176.5–1756.6 ng/g reported in a previous study on horses [15]. These drugs are known to have a large volume of distribution, which is a desirable characteristic in a fast-acting sedative. These drugs are also known to have relatively short half-lives and are rapidly excreted following administration in horses, cattle, sheep, and dogs [9,10]. Drug metabolism in exposed animals may produce some drug metabolites that were not evaluated in the present study because of the short time between administration and euthanasia.

Both drugs persisted through a simulated rendering process, and the concentrations were higher in the rendered tissues compared to their raw counterparts. The exception was xylazine in fat, which decreased slightly after rendering in the group receiving xylazine alone. Part of this increase can likely be attributed to moisture loss, as all tissue weights reduced by about 12% in weight after rendering. Importantly, in modern commercial rendering systems, moisture and fat are removed from solid cooked tissue during and immediately after cooking, respectively. The autoclave rendering model deployed in the present work would not allow moisture to fully escape, and the tissues were cooked in glass beakers, which did not allow liquid fat to be fully separated. Other studies have shown variable changes in oxytetracycline and tylosin concentrations in other matrices that were dependent upon the process used [26]. The composite sample’s weight reduced by about 17%, which may be because smaller 5 g pieces of each tissue were included, potentially making the moisture extraction more efficient than the 20 g samples due to increased surface area related to sample volume. The decrease in drug concentration in fat despite a 12.65% mean moisture loss may suggest that xylazine in fat is reduced by the rendering process. Importantly, the concentration of xylazine in fat after rendering is not zero and would exceed the 1 ng/g applicability threshold for USDA-FSIS screening.

The concentrations in rendered tissue may be considered in the context of the doses needed for sedation in species that may be fed rendered product. Oral doses of 2.5 mg/kg xylazine and 10 mg/kg ketamine were used concurrently in cats to evaluate their sedative effects in a previous study [17]. These doses were used as target doses, along with the highest detected rendered tissue concentrations (6866.80 ng/g xylazine in the kidney and 4528.20 ng/g ketamine in the kidney), in the present study to crudely represent the highest possible exposure. A 10-pound (4.54 kg) cat would need to ingest 11.36 mg of xylazine to reach the 2.5 mg/kg dose, which translates to 3.64 pounds (1.66 kg) of rendered bovine kidney containing 6866.8 ng/g xylazine in one sitting. The 10 mg/kg ketamine dose results in a calculated exposure of 22.2 pounds (10 kg) of rendered bovine kidney containing 4528.2 ng/g ketamine. This represents 36.4% and 222% body weight, respectively; both scenarios are not physiologically likely for an animal that consumes 2–4% body weight per day. The dosage calculations were performed based on concurrent dosing, individual dosing of each drug alone may result in differing levels of sedation. While toxicity is unlikely, inadvertent inclusion of sedative drugs in rendered products should be avoided.

The parent compounds xylazine and ketamine were found in the tissues of cattle following administration during euthanasia, followed by a simulated rendering process. Confirmation of these findings should be carried out in large-scale commercial rendering facilities. Differences between rendering procedures and individual facility components may affect the detection of these drugs. Drug metabolites were not evaluated in the present work; future work determining the rate at which these drugs are metabolized in a euthanasia scenario and the potential metabolites produced is needed. Future research detailing the oral bioavailability of these drugs in animals consuming food that utilizes rendered product is also warranted.

## Figures and Tables

**Table 1:** Individual and mean (SD) xylazine and ketamine concentrations (ng/g) from individual and composite raw and rendered bovine tissue samples (*n* = 6/treatment group).

Treatment Group	Animal 1	Animal 2	Animal 3	Animal 4	Animal 5	Animal 6	Mean (SD)
**Group 1 Xyl. Raw**							
Muscle	222.4	129.6	142.4	99.4	109.8	138.5	140.35 (43.51)
Fat	152.0	47.8	52.4	56.5	72.7	42.4	70.63 (41.17)
Liver	288.0	457.8	251.7	345.6	167.0	213.6	287.28 (103.58)
Kidney	1562.0	506.7	1308.5	749.3	1180.5	898.9	1034.32 (388.06)
Composite	558.6	291.1	433.0	384.9	398.0	364.0	404.93 (88.86)
**Group 1 Xyl. Rendered**							
Muscle	303.0	172.0	181.9	123.0	141.4	162.8	180.68 (63.61)
Fat	121.6	52.1	57.3	58.5	76.6	49.6	69.28 (27.32)
Liver	378.2	828.4	318.1	451.5	212.5	268.3	409.50 (221.50)
Kidney	2033.5	836.1	1747.3	1401.6	1823.9	1514.7	1559.52 (419.55)
Composite	649.8	414.7	535.0	354.0	422.6	373.6	458.28 (112.95)
**Group 2 Xyl. Raw**							
Muscle	409.5	66.5	29.7	489.3	261.1	568.3	304.07 (223.07)
Fat	68.9	20.3	6.2	95.4	40.8	192.2	70.63 (67.78)
Liver	754.4	31.8	16.2	543.5	538.7	641.3	420.98 (317.45)
Kidney	3629.0	657.0	190.7	2858	2979.7	4093.6	2401.33 (1602.72)
Composite	1320.7	169.0	71.2	1885.3	1289.2	1847.3	1097.12 (798.10)
**Group 2 Xyl. Rendered**							
Muscle	509.3	82.3	41.0	597.6	339.0	629.8	366.50 (257.14)
Fat	63.3	20.3	6.3	77.3	34.9	218.5	70.10 (77.34)
Liver	1087.7	35.8	20.4	192.1	715.3	834.0	480.88 (456.38)
Kidney	4633.9	914.5	251.0	6866.8	3729.5	5525.8	3653.58 (2602.96)
Composite	1573.2	214.9	73.8	2271.8	1096.2	1746.4	1162.72 (874.96)
**Group 2 Ket. Raw**							
Muscle	1474.2	156.5	328.9	1653	1028.2	1827.3	1078.02 (701.69)
Fat	215.5	52.6	44.8	461.2	160.8	1214.4	358.22 (446.01)
Liver	2261.1	66.2	65.4	819.3	971.4	1401.7	930.85 (836.90)
Kidney	2591.6	232.0	165.7	1304.9	1442.2	3321.3	1509.62 (1259.98)
Composite	1591.9	140.5	166.3	1808.9	850.4	1969.8	1087.97 (819.14)
**Group 2 Ket. Rendered**							
Muscle	1995.2	186.0	499.6	2340.8	1031.0	2247.6	1383.37 (935.61)
Fat	292.4	74.0	56.6	477.1	161.1	1624.3	477.58 (597.37)
Liver	2790.2	93.4	102.1	2661.4	1345.4	2087.8	1513.38 (1209.50)
Kidney	3528.2	316.2	235.1	3884.2	1840.3	4528.2	2388.70 (1862.60)
Composite	1930.8	162.6	210.3	2174.3	977.6	2421.6	1312.87 (1000.44)

**Table 2:** Mean (SD) percent change in individual xylazine and ketamine concentrations between raw and rendered cattle tissue samples (*n* = 6/treatment group).

Treatment Group	Muscle	Fat	Liver	Kidney	Composite
Group 1 Xylazine	24.83 (9.00)	−4.38 (11.92)	13.48 (39.62)	49.82 (44.59	8.33 (16.58)
Group 2 Xylazine	27.79 (6.60)	4.04 (12.65)	37.02 (21.64)	56.47 (21.79)	13.86 (17.79)
Group 2 Ketamine	28.50 (18.37)	23.35 (17.33)	72.15 (74.61)	62.65 (66.30)	20.26 (4.37)

**Table 3:** Mean (SD) percent change in sample weight attributed to moisture loss during the simulated rendering process (*n* = 12).

Tissue	Muscle	Fat	Liver	Kidney	Composite
% Change	−11.52% (1.82)	−12.65% (3.96)	−12.43% (2.20)	−11.90% (1.20)	−16.85% (2.19)

## Data Availability

The funders had no role in the design of this study; in the collection, analyses, or interpretation of data; in the writing of the manuscript; or in the decision to publish the results.

## Supplementary Materials

The following supporting information can be downloaded at https://www.mdpi.com/article/10.3390/vetsci12080740/s1: Figure S1: Individual group 1 xylazine concentrations in raw and rendered bovine tissues (*n* = 6). ● = raw tissue; ∆ = rendered tissue; Figure S2: Individual Group 2 xylazine concentrations in raw and rendered bovine tissue (*n* = 6). ● = raw tissue; ∆ = rendered tissue; Figure S3: Individual group 2 ketamine concentrations in raw and rendered bovine tissues (*n* = 6). ● = raw tissue; ∆ = rendered tissue. A supplementary file is included, graphically representing the data presented in Table 1.
