# Supplementary material for: Determining the Persistence of Xylazine and Ketamine in Cattle Tissue Following a Simulated Rendering Process
Source: Vet Sci. 2025 Aug 7;12(8):740. doi: 10.3390/vetsci12080740 (PMC12390323; doi:10.3390/vetsci12080740)
Supplement: Supplementary file 1 [file vetsci-12-00740-s001.zip › vetsci-3722801-supplementary.pdf]

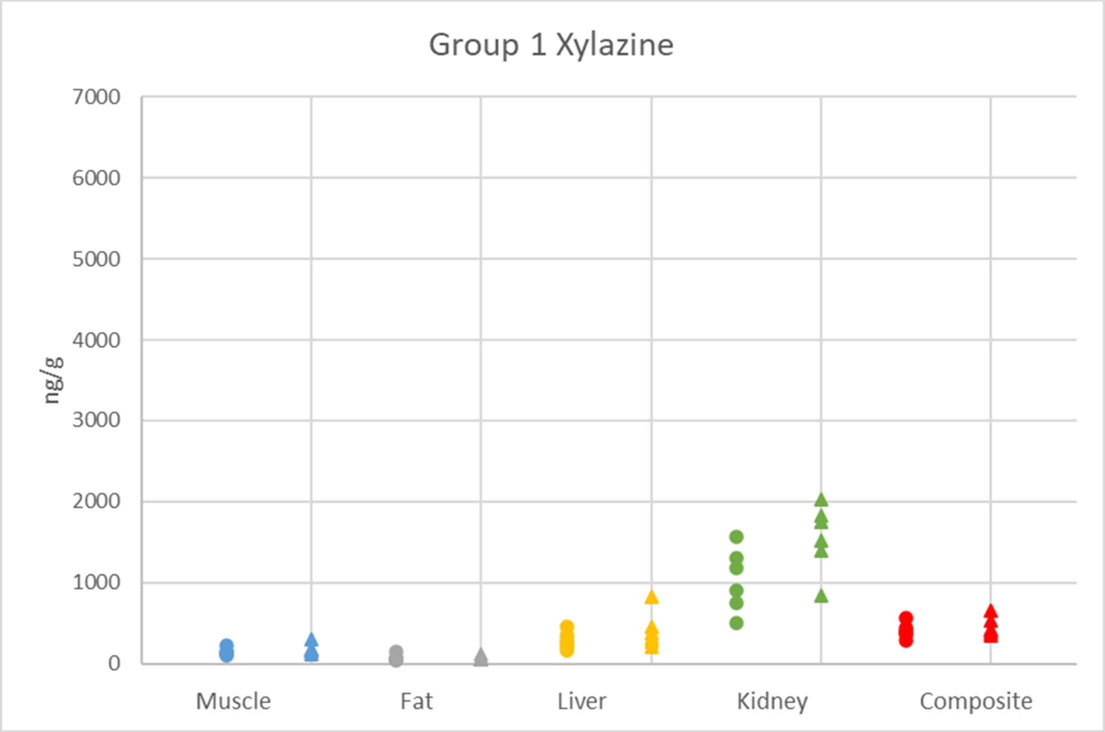

Figure S1. Individual Group 1 Xylazine concentrations in raw and rendered bovine tissue (n=6). ● = Raw Tissue Δ = Rendered Tissue.

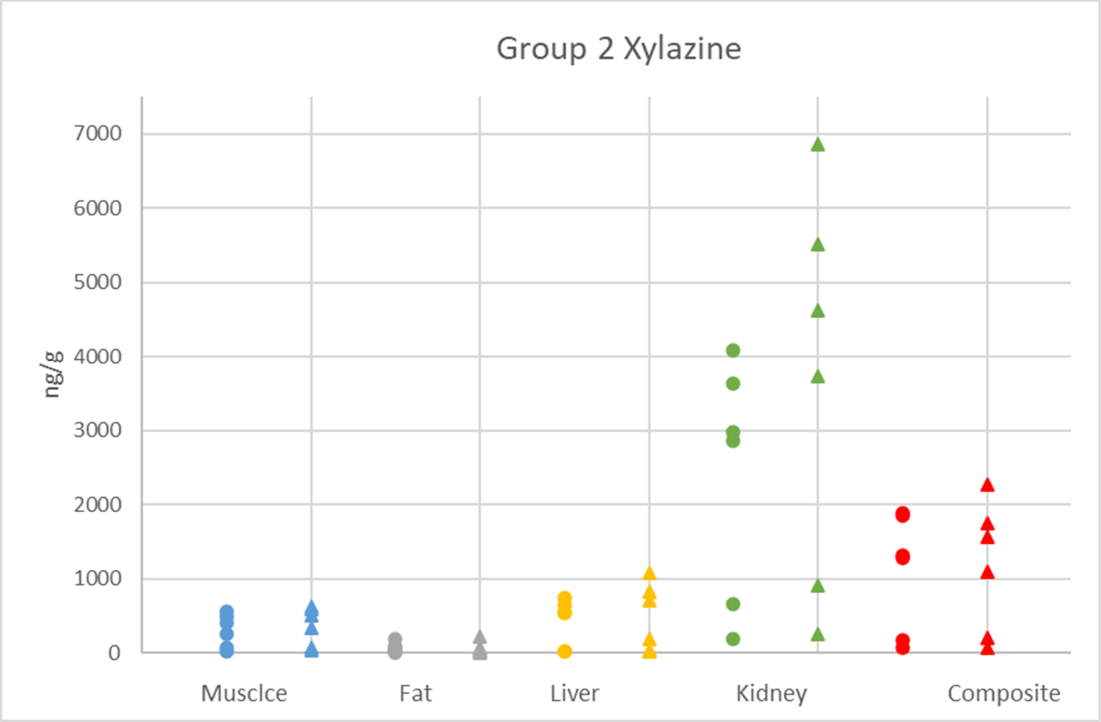

Figure S2. Individual Group 2 Xylazine concentrations in raw and rendered bovine tissue (n=6). ● = Raw Tissue Δ = Rendered Tissue.

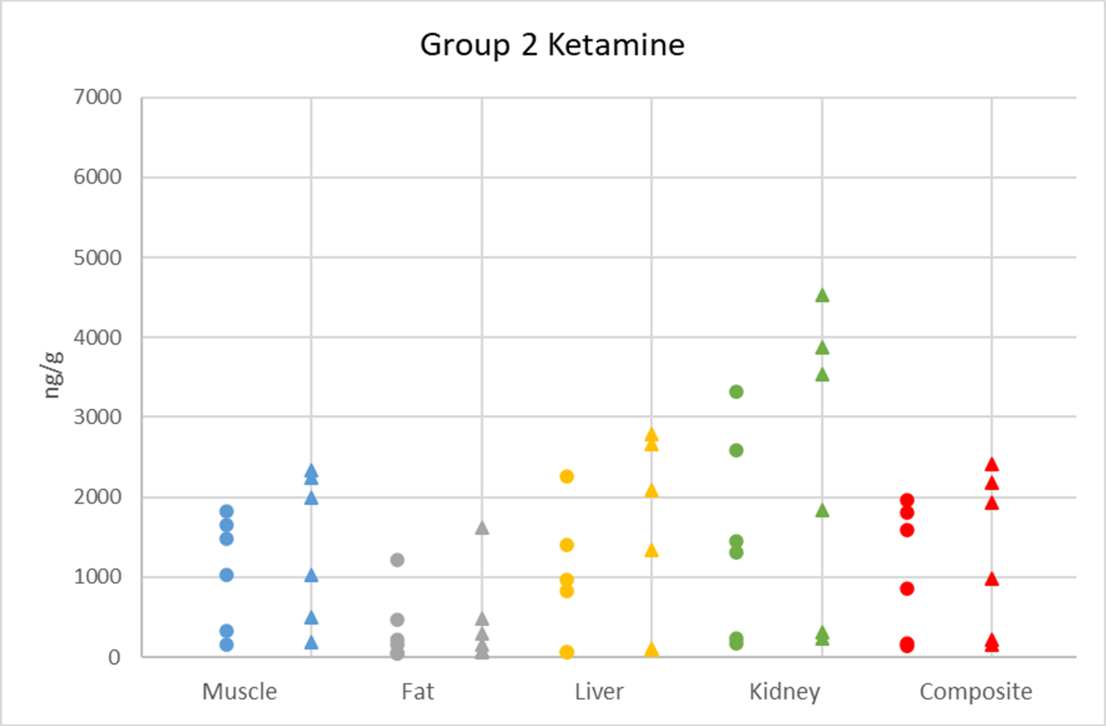

Figure S3. Individual Group 2 Ketamine concentrations in raw and rendered bovine tissue (n=6). ● = Raw Tissue Δ = Rendered Tissue.
